# Extracellular cystine influences human preadipocyte differentiation and correlates with fat mass in healthy adults

**DOI:** 10.1007/s00726-021-03071-y

**Published:** 2021-09-14

**Authors:** Hagar Elkafrawy, Radwa Mehanna, Fayrouz Ali, Ayman Barghash, Iman Dessouky, Fredrik Jernerén, Cheryl Turner, Helga Refsum, Amany Elshorbagy

**Affiliations:** 1 Department of Medical Biochemistry, Faculty of Medicine, Alexandria University, Alexandria, Egypt; 2 Center of Excellence for Research in Regenerative Medicine and Applications (CERRMA), Faculty of Medicine, Alexandria University, Alexandria, Egypt; 3 Department of Physiology, Faculty of Medicine, Alexandria University, Alexandria, Egypt; 4 Department of Pharmacology, University of Oxford, Oxford, UK; 5 Department of Pharmaceutical Biosciences, Uppsala University, Uppsala, Sweden; 6 Department of Nutrition, Institute of Basic Medical Sciences, University of Oslo, Oslo, Norway

**Keywords:** Cystine, Adipogenesis, Obesity, BMI, Sulfur amino acids

## Abstract

Plasma cysteine is associated with human obesity, but it is unknown whether this is mediated by reduced, disulfide (cystine and mixed-disulfides) or protein-bound (bCys) fractions. We investigated which cysteine fractions are associated with adiposity in vivo and if a relevant fraction influences human adipogenesis in vitro. In the current study, plasma cysteine fractions were correlated with body fat mass in 35 adults. Strong positive correlations with fat mass were observed for cystine and mixed disulfides (*r* ≥ 0.61, *P* < 0.001), but not the quantitatively major form, bCys. Primary human preadipocytes were differentiated in media containing cystine concentrations varying from 10–50 μM, a range similar to that in plasma. Increasing extracellular cystine (10–50 μM) enhanced mRNA expression of *PPARG2* (to sixfold**)**, *PPARG1*, *PLIN1*, *SCD1* and *CDO1* (*P* = 0.042– < 0.001). Adipocyte lipid accumulation and lipid-droplet size showed dose-dependent increases from lowest to highest cystine concentrations (*P* < 0.001), and the malonedialdehyde/total antioxidant capacity increased, suggesting increased oxidative stress. In conclusion, increased cystine concentrations, within the physiological range, are positively associated with both fat mass in healthy adults and human adipogenic differentiation in vitro. The potential role of cystine as a modifiable factor regulating human adipocyte turnover and metabolism deserves further study.

**Supplementary Information:**

The online version contains supplementary material available at 10.1007/s00726-021-03071-y.

## Introduction

The past decade has seen increasing focus on the associations of specific amino acids with adiposity and cardiometabolic outcomes. Excess intake of the essential sulfur amino acid (SAA) methionine is associated with BMI (Virtanen et al. 2006), diabetes and cardiovascular disease (CVD) (Virtanen et al. 2006; Dong et al. 2019; Tharrey et al. 2020). Although cysteine is the limiting constituent of the major antioxidant glutathione (Wu et al. 2004), plasma total cysteine (tCys) concentrations are associated with body total fat mass in adults and children of different ethnicities (Elshorbagy et al. 2008, 2012d), and predict 2 year weight regain following bariatric surgery (Hanvold et al. 2018). High cystine intake in mice lowered energy expenditure, induced the adipogenic transcription factor peroxisome proliferator-activated receptor-γ (*Pparg*) in adipose tissue and increased weight gain and visceral fat (Elshorbagy et al. 2012a). Conversely, restricted intake of the cysteine precursor methionine lowers tCys (Elshorbagy et al. 2010), raises energy expenditure, lowers adiposity (Hasek et al. 2010) and improves metabolic health in rodents (Yu et al. 2018) and humans (Plaisance et al. 2011). Cysteine supplementation reverses the anti-obesity (Elshorbagy et al. 2011) and antioxidant effects of methionine restriction (Gomez et al. 2015). Collectively, this evidence links plasma total cysteine to obesity but the possible mechanisms have not been elucidated in humans.

Over 60% of plasma cysteine is protein-bound (bCys) (Mansoor et al. 1992). Free cysteine is present in reduced (rCys) or oxidized forms, the latter including homogeneous (cystine) and mixed disulfides. Although cystine and rCys are inter-convertible, cystine is the more abundant form in the pro-oxidant plasma milieu. A more oxidized plasma cysteine redox state is associated with ageing, BMI and CVD (Patel et al. 2016; Oliveira and Laurindo 2018). In adults, the association of plasma tCys with fat mass is observed across the entire range of BMI, from normal weight to obese (Elshorbagy et al. 2008), but the association of each tCys component with fat mass has not been systematically investigated.

Human obesity is characterized by an increase in number as well as size of adipocytes (Stephens 2012). Adipogenesis occurs in two stages; commitment of mesenchymal stem cells to preadipocytes, and terminal differentiation of preadipocytes to mature adipocytes (Cristancho and Lazar 2011). The latter stage is induced by several adipogenic stimuli via activation of PPARγ, a key transcription factor which regulates genes responsible for cellular uptake, activation, transport and packaging of fatty acids into lipid droplets (Lefterova et al. 2008). Adding oxidised glutathione early during differentiation of human preadipocytes increased final lipid accumulation (Jones et al. 2016), but the effect of modulating extracellular cyst(e)ine on human adipogenesis is unknown. In 3T3-L1 cells, a mouse-derived cell line, increasing extracellular cystine concentrations enhanced *Pparg* expression and adipogenic differentiation and lipid accumulation (Haj-Yasein et al. 2017). While this provides a plausible mechanism by which cysteine could promote obesity, the interspecies differences in gene expression and adipogenic regulation between murine and human adipocytes render it problematic to extrapolate directly from 3T3-L1 data to human obesity (Mikkelsen et al. 2010; Schmidt et al. 2011).

Some links between the cysteine catabolic enzyme cysteine dioxygenase (CDO) and adipogenesis/fat gain have been reported. CDO initiates taurine synthesis from cysteine, although we have previously observed that plasma concentrations of tCys do not correlate with taurine (Elshorbagy et al. 2012b). In 3T3-L1 cells, *Cdo* expression increased six– to ninefold during differentiation to adipocytes (Tsuboyama-Kasaoka et al. 2006). In vivo, CDO is one of the most nutrient-responsive enzymes (Stipanuk et al. 2009). In mice, *Cdo* expression is high in adipose tissue (Ueki and Stipanuk 2009) and is markedly upregulated in response to increased dietary protein/cysteine (Stipanuk et al. 2009). It is not known whether *CDO *is induced during differentiation of human preadipocytes, and whether the degree of induction is dependent on extracellular cystine.

The present study sought to answer two questions: which fraction(s) of circulating cysteine are associated with adiposity in healthy adults; and whether physiologic concentrations of a relevant cysteine fraction can influence human adipogenic differentiation in vitro.

## Materials and methods

### Human study

#### Subjects

Cross-sectional associations of fasting plasma oxidized, reduced and protein-bound thiol fractions with body composition were evaluated using data from acid-precipitated plasma from *N* = 35 subjects (12 men and 23 women) who had given informed consent, after a detailed explanation of the procedures. The study was approved by the Ethics Committee of the Faculty of Medicine, Alexandria University (IRB code:00012098, FWA no:0001869, Serial no:0201071). Population characteristics and plasma fatty acid and amino acid biomarker profile were reported previously (Elshorbagy et al. 2017; Alshahawy et al. 2021). Briefly, this was a predominantly sedentary young adult population. Exclusion criteria comprised pregnancy, weight loss of > 2 kg over the month preceding baseline sampling and data collection, chronic renal or liver insufficiency, intake of medication known to affect body composition (e.g., corticosteroids), and regular physical exercise (defined as undertaking any of the following at least once per week for ≥ 30 min: exercising at the gym, swimming or engaging in any outdoor exercise activity, including game sports, running, or walking). Participants included 12 men and 23 women, aged (mean ± SEM: 29 ± 1.6 and 32 ± 2.4 year, respectively). None of the participants used lipid-lowering medication or had diabetes or CVD, although two were taking antihypertensive drugs. For the present analysis, baseline data (week 0) is used for the cross-sectional analysis.

#### Anthropometric data and blood sampling

Details of data collection were described previously (Elshorbagy et al. 2017). Body weight and composition were measured in light clothing using a whole-body bioelectrical impedance analysis (BIA) analyzer (InBody 220, Biospace, Korea). Waist circumference was measured at the end of expiration at a point mid-way between the lower rib margin and iliac crest. Hip circumference was measured as the greatest circumference around the buttocks, and the waist–hip ratio was used as marker of abdominal obesity. Overnight-fasted blood samples were collected in EDTA-lined vacuum tubes chilled on ice. Immediately, blood was centrifuged for 30 s, and 200 µL of the plasma supernatant was added to 600 µL 4% v/v perchloric acid and re-centrifuged for 2 min. The supernatant was used for assay of thiol fractions. The remaining plasma was re-centrifuged and used for assay of total SAAs and clinical biochemistry parameters.

#### Amino acid and clinical biochemistry assays

SAAs were assayed by liquid chromatography-tandem mass spectrometry (LC–MS/MS) using a Prominence LC-20AD XR binary pump (Shimadzu, Kyoto, Japan) coupled to a QTRAP 5500 hybrid triple quadropole mass spectrometer (AB Sciex, Framingham, MA, US). Plasma tCys, total homocysteine (tHcy), and total glutathione (tGSH) were analysed using a modification of a previously described method (Refsum et al. 2004), whereas taurine was extracted and assayed separately as described (Elshorbagy et al. 2017). SAM, SAH, rCys, rGSH, cystine, as well as the free (non-protein bound) fractions of cysteine, homocysteine and glutathione were extracted from PCA-treated plasma using the same protocol and conditions as for the tCys assay (Refsum et al. 2004), adjusted for the dilution of the samples. Quantitation of all analytes was based on comparison with standard curves corrected for presence of isotopically labelled internal standards using a 1/x weighting. % coefficient of variation for all amino acid analyses were < 10%. Protein-bound concentrations of the three thiols were calculated as the total minus free concentration of the relevant thiol. Cysteine mixed disulfides were calculated as non-protein bound cysteine minus the sum of rCys and cystine. Plasma albumin and total protein were spectrophotometrically measured at 505 nm, by a colorimetric assay as described (Doumas et al. 1981).

### In vitro study

#### Human adipose tissue samples

All in vitro experiments were conducted at Center of Excellence for Research in Regenerative Medicine and its Application (CERRMA), University of Alexandria, Egypt, using primary cultures of cells derived from the stroma-vascular fraction (SVF) of human adipose tissue. Subcutaneous adipose tissue lipoaspirate (50 mL) was obtained during elective abdominal liposuction procedures performed at Alexandria University Hospitals from five women who had given written informed consent, after a detailed explanation of the procedure. The study was performed in accordance with the ethical standards laid down in the 1964 Declaration of Helsinki and its later amendments and was approved by the Ethics Committee of the Faculty of Medicine, Alexandria University (IRB code:00012098, FWA no:0001869, Serial no:0201071). The donors were aged 26–42 year with a BMI of 25–30 kg/m^2^ and a waist to height ratio of 0.45–0.59. The donors were selected to be free of chronic disease as assessed by medical history and routine laboratory tests and were not on oral contraceptive, lipid-lowering or steroid medication. They were not pregnant or breast-feeding.

#### Reagents

Cell culture reagents were obtained from Sigma-Aldrich (St. Louis, MO, US). Tissue culture plastics were from Corning Incorporated Life Sciences (Corning, NY, US); please see Supporting Information Table S1 for details.

#### Isolation and culture of adipocyte precursor cells

The protocol for isolation and culture of adipocyte precursor cells was modified from Bunnell et al. (2008) and is detailed in the Supporting Information. After isolation, cells were counted, seeded into 12-well plates in growth media (DMEM 4.5 g/L glucose with l-glutamine, 10% fetal bovine serum, 1% antibiotics (10,000 IU/mL penicillin, 10,000 μg/mL streptomycin), and incubated at 37 °C/5% CO_2_. Cell growth and proliferation were monitored daily using the contrast phase inverted microscope, and the media changed every 2 days. After 6–8 days of proliferation, when cells reached 75–80% confluence, the adipocyte differentiation protocol with variable cystine concentrations was started.

#### Differentiation of adipocyte precursor cells under varying cystine concentrations

For the preadipocyte differentiation study, methionine- and cysteine-deplete DMEM (Sigma-Aldrich #D0422) supplemented with 30 μM l-methionine (Sigma-Aldrich #M5308) and variable concentrations (10, 15, 30, or 50 μM) of l-cystine (Sigma-Aldrich #C7602) from individual stock solutions of l-methionine (20 mM) dissolved in H2O and l-cystine (10 mM) dissolved in 0.2 M HCl, were used. The concentration of methionine (30 μM) was selected based on previous studies in 3T3-L1 adipocytes (Haj-Yasein et al. 2017) and was comparable to the plasma methionine concentrations observed in the present human study (median 25.2 μmol/L in men and 20.2 μmol/L in women). To induce differentiation, cells were treated (on day 0) with an induction medium (Bunnell et al. 2008) (containing 1 μM dexamethasone, 58 μg/mL insulin, 0.5 mM 3-isobutyl-1-methylxanthine, and 200 μM indomethacin) and media was changed with fresh induction media on day 2. On day 4 cells were treated with insulin medium (containing 10 μg/mL insulin), and medium was changed every 2 days till day 8, when clusters of mature adipocytes filled with lipid droplets were visible.

In pilot experiments, we also tested the effect of regular DMEM (Sigma-Aldrich, #D6546). Regular DMEM contains 200 μM l-methionine (0.03 g/L) and 200 μM l-cystine HCL (0.0626 g/L). Regular DMEM had a similar effect on differentiation to that of methionine- and cysteine- deficient DMEM supplemented with 50 μM of cystine and 30 μM of methionine, as assessed by morphologic examination of Oil Red *O* stained adipocytes and quantification of their lipid content (Supporting information Fig. S1). Total absence of cystine in culture media resulted in cell death after 1–3 days of shifting to the cystine-free differentiation medium.

#### Assessment of cell viability

The methylthiazolyldiphenyl-tetrazolium bromide (MTT) assay (Mosmann 1983) was used to evaluate the effect of different cystine concentrations on adipocyte viability on day 8 of differentiation. Mitochondrial enzymes in viable cells reduce MTT to formazan crystals, which are dissolved by DMSO and the resulting coluored solution was spectrophotometrically measured at 570 nm.

#### Reverse transcription quantitative polymerase chain reaction (RTqPCR)

##### Isolation and reverse transcription of RNA

On day 0 and day 4 of differentiation, cells were washed twice with ice-cold PBS, lysed by 500 µL Qiazole and frozen at − 80 °C until RNA isolation. Total RNA was isolated by a spin protocol (Qiagen RNeasy Mini Kit #74104). RNA concentrations and quality (260/280 ratio) were determined on a Thermo Scientific ND2000 Nanodrop Spectrophotometer and stored at − 80 °C. Total RNA (12.5–25 ng/μL) was reverse transcribed using the high-capacity cDNA reverse transcription kit (Life Technologies #4374966) on an Applied Biosystems GeneAmp PCR System 9700 N8050200 thermal cycler with the following settings: 25 °C for 10 min, 37 °C for 120 min, 85 °C for 5 s, and 4 °C on hold. cDNA was stored at − 20 until qPCR experiments.

##### qPCR and data quantification

Gene-specific regions were amplified from cDNA (5–10 ng/μL) with assay primers (100 nM each; Biosearch Technologies (Novato, CA, US)) and Maxima SYBR Green/ROX kit (Thermo Scientific #K0221) on an Applied Biosystems^™^ StepOne^™^ Real-Time PCR System 4376357 with the following settings: 25 μL reaction, 95 °C for 10 min, followed by 40 cycles; 95 °C for 15 s, 60 °C for 30 s and 72 °C for 30 s. Gene expression analysis was performed with Stepone Applied Biosystems software using the relative quantification (ΔΔCt) method. Results are presented as fold change relative to β-actin (2 − ΔΔCt). Primer sequences are listed in Supporting Information Table S2.

#### Oil Red O triglyceride staining

To assess lipid accumulation, Oil Red *O* staining was performed on differentiated mature adipocytes on day 8 of differentiation as described. (Ramírez-Zacarías et al. 1992) At least five images per well were taken with a camera-equipped inverted microscope (Olympus CKX41) at 200 × magnification and analysed for mean lipid droplet size and percentage of lipid-stained area (Kim et al. 2006) using Fiji image analysis software (NIH, Bethesda, USA).

#### Measurement of oxidative stress index (MDA/TAC index)

On day 0 and day 8 of differentiation, oxidative stress status was indirectly assessed by measuring the ratio of the lipid peroxidation product malondialdehyde (MDA) to total antioxidant capacity (TAC) (MDA/TAC oxidative stress index) (Badehnoosh et al. 2018) in the culture media at different cystine concentrations. MDA was measured using the thiobarbituric acid (TBA) spectrophotometric method (Ohkawa et al. 1979), based on the reaction of MDA and TBA under high temperature, forming a coloured adduct measured at 534 nm. TAC was measured by calorimetry based on the reaction of antioxidants in the sample with exogenously provided H_2_O_2_, with generation of a chromophore measured at 505 nm (Koracevic et al. 2001). MDA/TAC ratio was obtained by dividing the values of MDA (µmol/L) by TAC (mmol/L).

### Statistical analysis

For the human study, due to skewness of several analytes, non-parametric analysis was used. Population characteristics are presented as median (25th, 75th percentiles) or %, and gender comparisons were conducted by Mann Whitney *U* test or Chi-squared test, respectively. Correlations were assessed by the Spearman rank correlation coefficient with adjustment for relevant covariates. For the in vitro study, data are presented as mean ± SEM and compared across groups by one-way ANOVA. All tests were two-tailed. *P* ˂0.05 was considered statistically significant, and *P* < 0.1 was discussed as a trend. PASW Statistics for Mac (20.0; SPSS Inc., Chicago, IL, USA), the R statistical environment (version 3.5.0 for Mac) and GraphPad Prism (version 8.3.1. for Windows) were used for data analysis.

## Results

### Characteristics and thiol concentrations in the study population

The study population comprised predominantly young, non-smoking men (*N* = 12) and women (*N* = 23) (Elshorbagy et al. 2017). Selected characteristics and plasma SAA and thiol profile are shown in Table 1. Median BMI was 28 kg/m^2^, and 58% of women and 75% of men were overweight or obese. Women had lower concentrations of methionine, cystathionine, tGSH and fGSH compared to men (Table S3). The distribution of plasma tCys in the total population was: bCys 62%, rCys 2.2%, cystine 15.5%, and mixed cysteine disulfides 20.3%.

**Table 1:** Population characteristics and plasma sulfur amino acid and thiol profile

	Men (*N* = 12)	Women (*N* = 23)
Age (years)	28.0 (25.5, 32.0)	29.0 (24.5, 32.5)
BMI	29.7 (25.4, 32.5)	27.21 (22.21, 33.51)
Overweight, BMI 25–29.9 (% of participants)	33	25
Obese, BMI ≥ 30 (% of participants)	42	33
Fat-free mass (kg)	61.1 (53.4, 62.7)	41.3 (35.9, 46.5)*
Fat mass (kg)	24.8 (19.8, 34.2)	30.2 (21.8, 41.9)*
Body fat (%)	30.2 (24.0, 37.9)	41.3 (35.8, 49.9)*
Waist/hip ratio	0.92 (0.90, 0.96)	0.95 (0.87, 1.03)
Plasma variables		
Total cysteine (µmol/L)	252 (233, 279)	258 (224, 278)
Reduced cysteine (µmol/L)	6.1 (4.95, 7.7)	5.3 (4.0, 6.9)
Cystine (µmol/L)	41.4 (36.2, 46.7)	40.3 (30.9, 48.2)
Mixed cysteine disulfides (µmol/L)	53.2 (47.8, 62.6)	51.1 (39.4, 57.9)
Bound cysteine (µmol/L)	155 (135, 164)	159 (130, 181)
Total homocysteine (µmol/L)	9.45 (8.50, 10.65)	7.80 (6.90, 9.35)
Free homocysteine (µmol/L)	1.70 (1.55, 1.95)	1.50 (1.20, 1.90)
Total glutathione (µmol/L)	8.62 (6.96, 9.32)	6.6 (5.95, 8.65)*
Reduced glutathione (µmol/L)	4.20 (3.55, 4.85)	3.30 (2.40, 4.10)
Free glutathione (µmol/L)	6.90 (5.80, 7.45)	5.30 (4.40, 6.50)*
Methionine (µmol/L)	25.2(22.4, 26.6)	20.2(17.4, 22.3)*
S-adenosyl methionine (nmol/L)	65.6 (58.9, 76.4)	66.4 (57.4, 77.5)
S-adenosyl homocysteine (nmol/L)	34.7 (25.6, 43.7)	25.1 (19.8, 39.2)
Cystathionine (nmol/L)	126 (110, 138)	92.8 (75.8, 111)*
Taurine (µmol/L)	91 (68, 128)	82 (59, 116)
Albumin (g/dL)	4.00 (3.75, 4.35)	4.10 (3.90, 4.20)
Total protein (g/dL)	6.90 (6.45, 7.25)	7.05 (6.65, 7.55)

Data are presented as median (25th, 75th percentile) or %, and compared by Mann–Whitney *U* test or Pearson’s *X*^2^ test

*P < 0.05 vs men

### Associations among plasma thiol forms and body composition

**Fig. 1:**
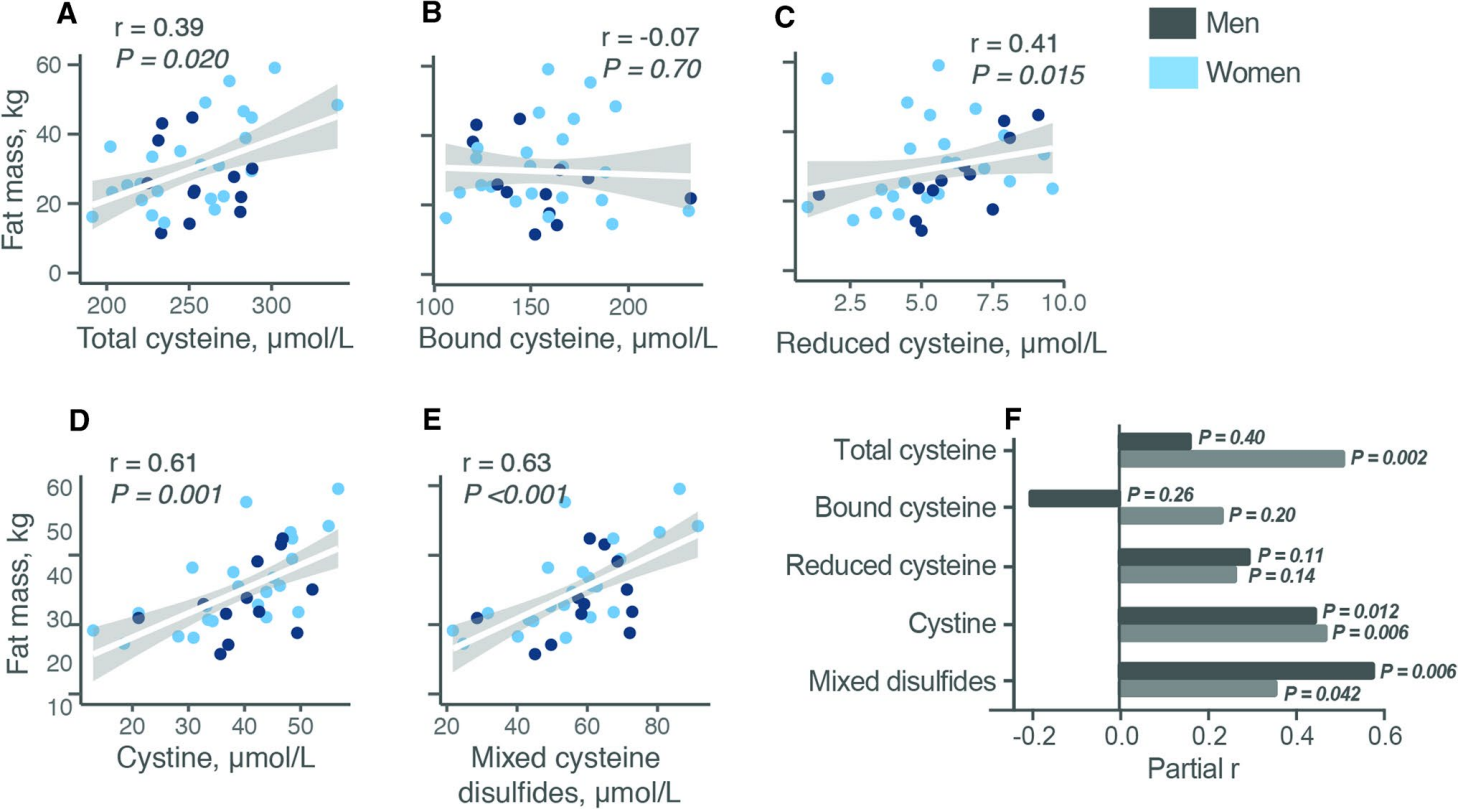
Correlation of different plasma cysteine species with age and fat mass. A–E Scatterplots and unadjusted Spearman correlation coefficients for the relation of different cysteine forms in fasting plasma with body total fat mass in adults; *N* = 35. **F** Partial correlations (Spearman) of cysteine forms with fat mass after adjustment for age, gender and fat-free mass (black bars) and with age after adjustment for gender (grey bars)

Scatterplots and unadjusted Spearman analysis showed strong positive correlations of cystine and mixed disulfides with fat mass (*r* ≥ 0.61, *P* < 0.001). tCys and rCys were also correlated with fat mass, whereas bCys was not (Fig. 1A–E). After adjustment for age, gender and lean mass, the only cysteine species associated with fat mass were cystine and mixed disulfides (Fig. 1F). These were also the two species that correlated with age. Cystine and mixed disulfides were also correlated with waist–hip ratio after adjustment for age and gender (partial *r* = 0.49 and 0.57, respectively, *P* ≤ 0.004), but rCys and bCys were not (*P* ≥ 0.48).

A cross-sectional Spearman correlation matrix among the aminothiols adjusted for age and gender is shown in (Supporting Information Table S2). All cysteine fractions, apart from rCys, correlated with tCys. rCys and rGSH were strongly correlated (partial *r* = 0.70). tHcy and tGSH and their components were not associated with age or adiposity. tCys, tHcy, tGSH and their fractions did not correlate with plasma albumin, total protein or other SAAs (methionine, SAM, SAH, cystathionine and taurine; data not shown) except for a negative correlation of tGSH with SAM, and positive correlations of tGSH with SAH and taurine. None of the SAAs or thiol components correlated with lean mass after adjusting for age, gender and fat mass (data not shown).

Since the free disulfide cysteine fractions were exclusively those associated with fat mass, the effect of cystine on human adipogenesis was investigated. Plasma cystine concentrations in the study population ranged from 13.0 to 56.7 μM. For the in vitro experiments, similar physiologic cystine concentrations of 10–50 μM were tested.

### Effects of extracellular cystine concentrations on adipogenic gene expression in human adipocyte precursors

**Fig. 2:**
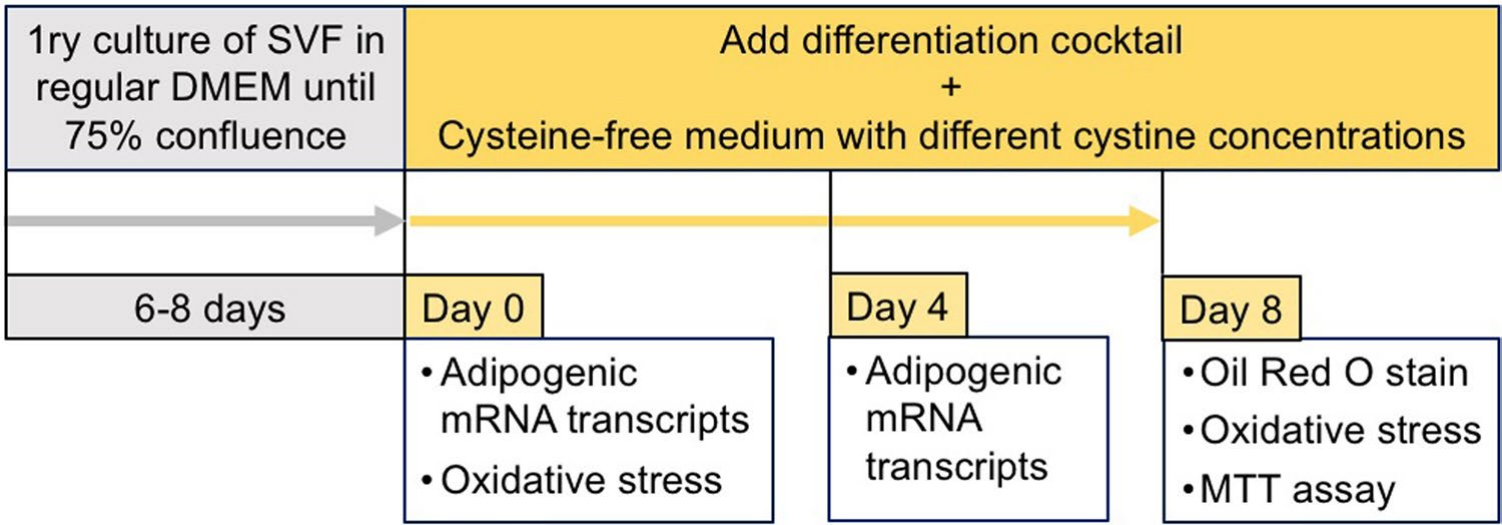
Schematic diagram of the timescale of the differentiation protocol (top) and outcome measures (bottom) of the human preadipocyte differentiation study (timepoints not to scale). *SVF* stromal vascular fraction. For details see Methods “In vitro study”

To evaluate the effect of cystine on human adipogenesis, we tested the mRNA expression levels of the adipogenic transcription factor PPARG and related genes in preadipocytes differentiated for 4 days under ascending cystine concentrations. The timescale of the proliferation and differentiation protocols and outcomes tested are shown in Fig. 2.

**Fig. 3:**
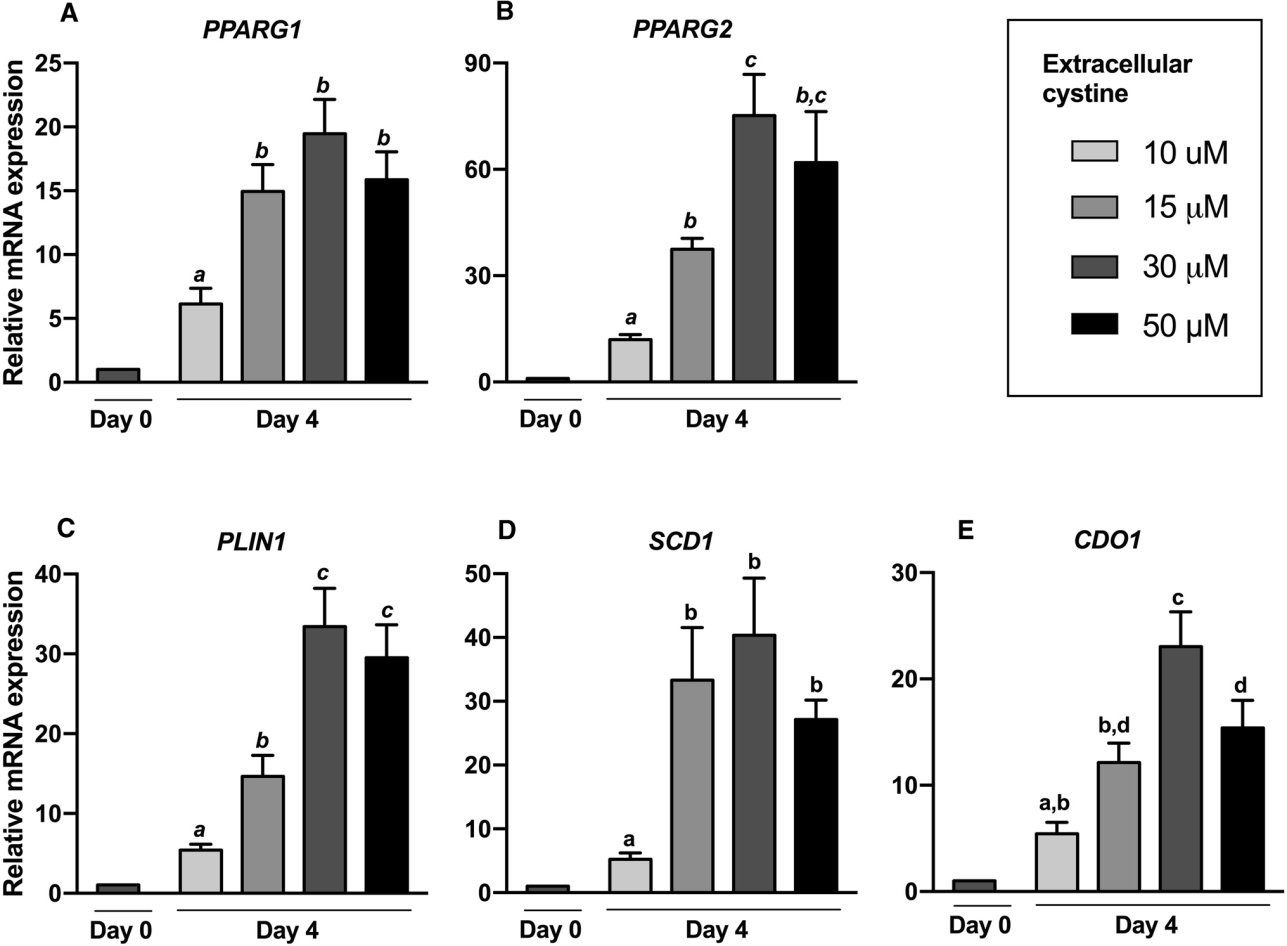
Relative mRNA expression of adipogenic genes and the cysteine catabolic enzyme cysteine dioxygenase1 (*CDO1*) on day 0 and day 4. Human preadipocytes were harvested on day 0 or differentiated in 10, 15, 30, or 50 μM cystine and harvested on day 4, then subjected to RT-qPCR testing. Results are presented as mean ± SEM from 5 independent experiments, each performed in triplicate. Bars not sharing the same letter are significantly different (*P* ˂0.05)

A severalfold induction on day 4 relative to day 0 of differentiation was observed for all adipogenic/lipid-related genes tested (all *P* < 0.001; Fig. 3A–D), with *PPARG2* showing the greatest relative induction. On day 4, the degree of induction of *PPARG2* and its target gene *PLIN1* at 15 μM and 30 μM cystine was, respectively, threefold and six–sevenfold that at 10 μM cystine (*P* < 0.05). There was no further increase at 50 μM cystine (Fig. 3B, C). In case of *PPARG1 *and *SCD1* there was, respectively, a 2.5- and sixfold higher expression at 15 vs 10 μM cystine (both pairwise comparisons *P* < 0.05), but no further increase at higher cystine concentrations (Fig. 3A, D). The cysteine catabolic enzyme *CDO1* was also induced during adipogenesis (*P* < 0.001), by 5- to 23-fold relative to day 0, in direct proportion to cystine concentrations, up to 30 μM cystine. A statistically significant 33% decrease in *CDO1* expression was observed at 50 μM relative to 30 μM cystine (Fig. 3E).

### Effects of extracellular cystine on adipocyte viability and lipid accumulation

**Fig. 4:**
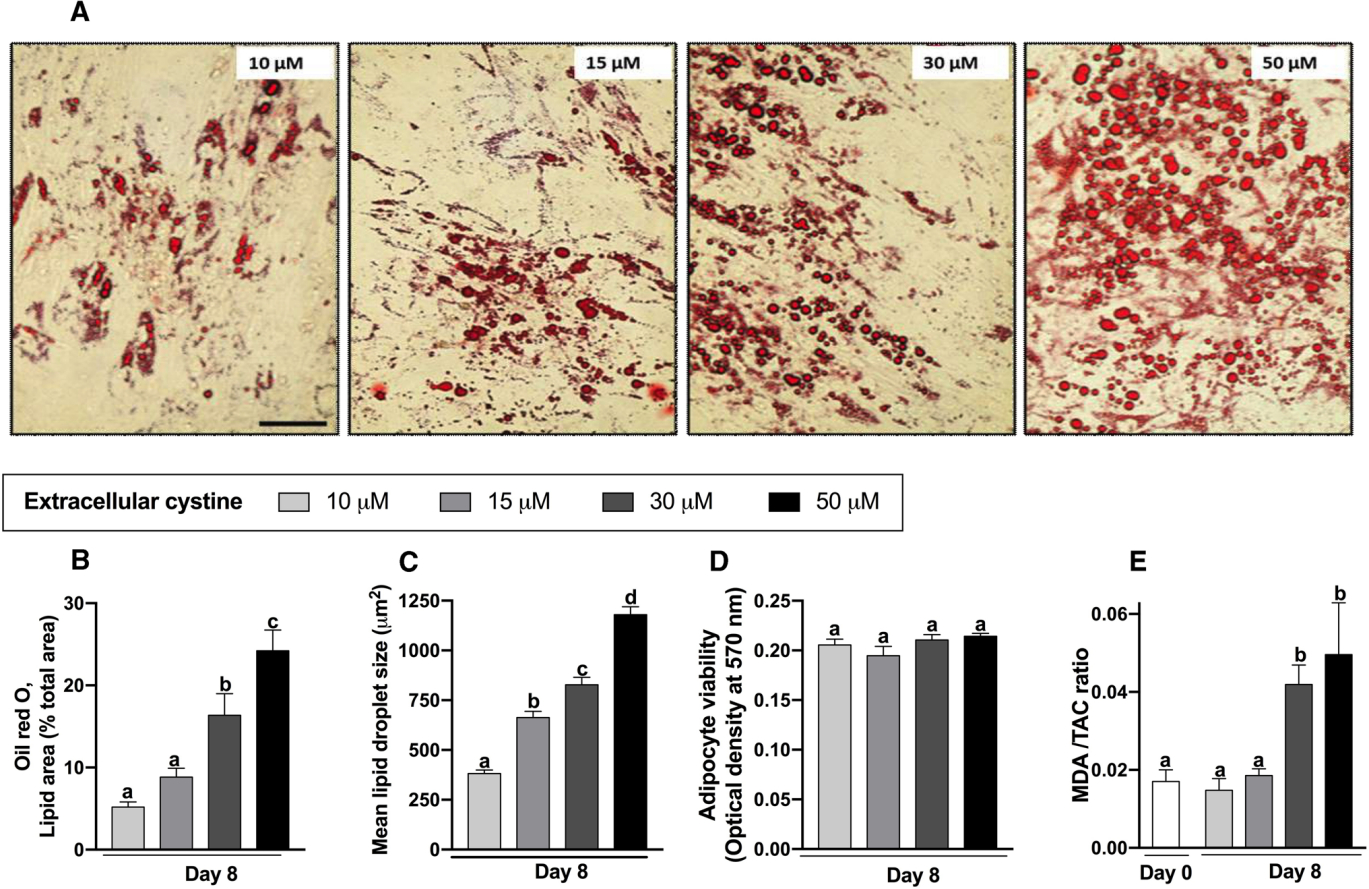
Lipid accumulation, cell viability and oxidative stress in differentiated mature adipocytes under ascending cystine concentrations of 10–50 μM. **A** Representative images of Oil Red *O* staining for lipid content in human adipocytes under varying cystine concentrations as shown; day 8 (magnification 200×; scale bar denotes 100 µm). **B** Quantification of the lipid-stained area on day 8. **C** Average lipid droplet size in differentiated adipocytes (**B** and **C** are based on 5 digital images/cystine concentration from three independent experiments, analysed using Fiji image analysis software (NIH, Bethesda, USA). **D** Cell viability of differentiated adipocytes assessed by the MTT assay. **E** Malonedialdehyde/total antioxidant capacity (MDA/TAC) oxidative stress index in culture media collected on day 0 and day 8 of differentiation. Results are mean ± SEM from 3–5 independent experiments, each performed in triplicate. Bars not sharing the same letter are significantly different (*P* ˂0.05)

Eight Days after initiation of differentiation, Oil Red *O*-stained lipid content was visibly greater in cells incubated with higher cystine concentrations compared to lower concentrations (Fig. 4A). Quantification of the percentage lipid area stained revealed a dose-dependent increase (*P* < 0.001) from 10 to 50 μM cystine (Fig. 4B), without the ceiling effect at 30 μM cystine that was observed for adipogenic gene expression. Lipid-droplet size also showed a dose-dependent increase (*P* < 0.001), up to 50 μM cystine (Fig. 4C). To evaluate the effect of cystine on the viability of mature adipocytes, the MTT assay was performed at late differentiation (day 8). No significant difference in the mean absorbance at different cystine concentrations was observed (Fig. 4D). Taken together, these findings indicate that human adipocytes tolerate all tested cystine concentrations but accumulate higher amounts of lipids when incubated with higher cystine.

### Oxidative stress as a function of extracellular cystine concentrations

Human studies report a correlation between fat accumulation and systemic oxidative stress (Liu et al. 2012). To assess oxidative stress at different cystine concentrations we used the MDA/TAC index (Badehnoosh et al. 2018), which measures the ratio of the end product of free-radical action on polyunsaturated fatty acids (MDA), to the antioxidant mechanisms protecting against this injury (TAC). The MDA/TAC ratio was not increased on day 8 of differentiation at lower cystine (10 and 15 μM) relative to day 0, but at 30 μM and 50 μM cystine it was approximately two-fold the pre-differentiation levels (Fig. 4E).

## Discussion

There is robust evidence from epidemiologic studies, animal models and murine adipocytes suggest that cyst(e)ine availability is related to adiposity (Elshorbagy et al. 2012a; Elshorbagy 2014; Haj-Yasein et al. 2017). Using fasting acid-precipitated plasma from healthy subjects we found that it is only the free disulfide fraction, including cystine that drives the association of plasma tCys with fat mass. In contrast, the quantitatively major protein-bound fraction is unrelated to adiposity. In primary culture of human preadipocytes, the cystine concentration in culture medium had a positive dose-dependent effect on adipocyte differentiation and lipid accumulation. These findings suggest that cystine, over the range typically present in plasma, may influence human adipogenesis. To our knowledge this is the first study linking physiologic extracellular concentrations of a nutrient/metabolite with both adiposity at the population level, and with human adipogenesis at the cellular level.

A key finding is that bCys, which in the present study population constituted 62% of tCys, is not associated with body fat mass. This suggests that it is not the total body cysteine pool, but rather a more oxidized cysteine redox state that is associated with obesity. In support of this, cystine/GSH ratio was found to increase with increasing BMI (Bettermann et al. 2018). Studies in 3T3L1 cells show that increasing the extracellular cystine at the expense of rCys while maintaining the tCys constant enhances adipogenic differentiation (Imhoff and Hansen 2010), whereas the antioxidant cysteine donor, N-acetylcysteine, inhibits adipogenesis (Calzadilla et al. 2011). The present study extends these findings to human cells, where increasing cystine in the culture medium at the start of human preadipocyte differentiation enhanced adipogenic differentiation and lipid peroxidation. Not surprisingly, a more oxidizing environment is present in visceral and subcutaneous fat from obese compared to lean individuals (Akl et al. 2017). The effect of other disulfides, namely homocystine and glutathione disulfide on adipogenesis deserve investigation.

The aim of the in vitro study was to characterize a model as close as possible to the cystine-fat mass association observed at the population level, using physiologic concentrations. Increasing extracellular cystine enhanced *PPARG1* and *PPARG2* expression in preadipocytes, and their differentiation to mature adipocytes, with an apparently greater effect of cystine on *PPARG2*. Among the different splice variants of *PPARG *gene in humans, PPAR-γ2 is exclusively expressed in adipocytes and is considered the main driver of human adipogenesis (Aprile et al. 2014). Cystine also had a dose–response effect on *PLIN1*, which coordinates lipolysis and lipid storage (Sztalryd and Brasaemle 2017). The effect of cystine on expression of the lipogenic enzyme *SCD1* featured a threshold effect from 10 μM relative to all higher concentrations, which is different from the linear and larger effect reported in murine 3T3L1 cells (Haj-Yasein et al. 2017).

We previously observed that adults featured *CDO1* mRNA induction in adipose tissue post-overfeeding, but only in those with higher fat mass gain (Elshorbagy et al. 2018). That adipogenesis itself is associated with *CDO1* induction independent of cystine concentrations is evidenced by the fivefold induction observed even at the lowest cystine concentration (10 μM) on day 4 relative to day 0 of differentiation, in line with findings in 3T3L1 cells (Stipanuk et al. 2009; Deng et al. 2015). Further, the degree of *CDO1* induction in human preadipocytes in the present study was cystine-dependent, reaching 23-fold induction at 30 μM cystine. Since CDO initiates taurine synthesis from cysteine, the CDO induction during adipogenesis is counterintuitive, given the anti-obesity effects of taurine in animal models (Murakami 2017). However, the taurine relationship with obesity in humans is more complex. Fasting plasma taurine is not associated with BMI in adults (Elshorbagy et al. 2012c), and a meta-analysis of 12 randomized controlled trials concluded that taurine supplements have no significant effect on BMI (Guan and Miao 2020). In light of this, the present findings suggest a role of CDO1 in human adipogenesis independent of taurine generation. In 3T3L1 cells, *Cdo1* was shown to play an essential role in the recruitment of PPAR-*γ* to the promoter of C/EBPα, another a key transcriptional mediator of early adipogenesis (Deng et al. 2015). In addition to CDO1, the cystine product hydrogen sulfide was shown to be required for cystine-mediated adipogenesis in 3T3L1 cells, suggesting that cystine availability triggers a host of downstream signals promoting adipogenesis.

Although hypertrophy of fat cells is the major cause of fat expansion in adults, hyperplasia also plays a role (Vishvanath and Gupta 2019). In obese subjects, the number of new adipocytes produced per year are nearly three-fold greater than that in lean individuals (Spalding et al. 2008). The molecular cues regulating human adipogenesis are largely not yet understood, but studies in mice show that it is the extracellular micro-environment, rather than intrinsic preadipocyte characteristics, that drive depot-specific adipogenesis in response to diet (Jeffery et al. 2016). Despite the clear stimulation of adipogenesis by high-physiologic cystine concentrations in the present study, an extrapolation to obesity causation in vivo cannot be attempted without taking into account how total body energy balance is affected. In this respect, we have previously shown that a high cystine intake in mice decreases resting energy expenditure and promotes weight gain (Elshorbagy et al. 2012b), although this is yet to be tested in humans.

Plasma cystine effects on *PPARG* induction and adipogenesis were demonstrated in the present study in abdominal subcutaneous preadipocytes. The transcriptional mechanisms leading to *PPARG* induction in subcutaneous preadipocytes are partly distinct from those in visceral preadipocytes (Vishvanath and Gupta 2019). Further, while subcutaneous gluteofemoral fat protects metabolic health (Manolopoulos et al. 2010), expansion of the subcutaneous abdominal and visceral fat depots is linked to insulin resistance and cardiac risk (Marinou et al. 2014; Vishvanath and Gupta 2019). In line with the positive effect of cystine on adipogenesis in subcutaneous abdominal cells, plasma cystine correlated with waist–hip ratio in the present study, and tCys has been associated with trunk/total fat ratio (Elshorbagy et al. 2013) and insulin resistance (Elshorbagy et al. 2012d). However, given the divergent regulation and metabolic consequences of adipogenesis in different depots (Vishvanath and Gupta 2019), the effect of cystine on visceral and gluteofemoral preadipocytes may differ from subcutaneous abdominal preadipocytes and remains to be tested.

Extensive control experiments in 3T3-L1 preadipocytes have shown that effect on adipogenesis is unique to cystine; valine, leucine, isoleucine, phenylalanine and histidine had a far weaker effect, or no effect, on expression of adipogenesis genes (Haj-Yasein et al. 2017). In the present study, although the cystine-dependency of induction of the main adipogenic gene, *PPARG2*, was capped at 30 μM, the adipocyte lipid accumulation and lipid droplet size showed dose-dependent increases up to 50 μM. This suggests that cystine may affect other aspects of adipocyte metabolism post-adipogenesis that enhance fat accumulation, including lipogenesis/lipolysis, as observed in primary mature rat adipocytes (Czech and Fain 1972; Olefsky 1979).

The main strength of the study is the simultaneous demonstration of a strong association of plasma cystine with fat mass in adults, and an effect on human adipogenesis of similar cystine concentrations. The primary preadipocytes were derived from volunteers of similar age, BMI and ethnicity to those in whom the cystine-fat mass association was demonstrated. Immediate acid precipitation of plasma, and use of deuterated cystine internal standard in the assay ensured accuracy of the human study measurements. The in vitro study was designed primarily for proof of concept, and more work is needed for elucidation of downstream mediators. Also, although an effect of cystine on adipogenesis was shown, it cannot be conclusively determined that the same occurs in vivo where metabolic, endocrine and paracrine cues could modify cystine effects on preadipocytes. Even if cystine influences adipocyte hyperplasia in vivo*,* the effect of cystine on lipid turnover in differentiated human adipocytes warrants investigation, given that hypertrophy plays a greater role in adult obesity than hyperplasia.

## Conclusions

In conclusion, fasting plasma concentrations of the free disulfide fractions of cysteine were strongly and independently associated with fat mass in healthy subjects, explaining the documented tCys association with adiposity in multiple cohorts. Notably, the quantitatively major protein-bound fraction was unrelated to adiposity. Increasing physiologic concentrations of cystine enhanced adipogenesis and lipid accumulation in primary culture of human adipose-derived stem cells in a dose-dependent manner. This provides proof of concept that extracellular cystine concentrations can influence adipogenesis, and one mechanism linking cystine to human obesity. Further studies are needed to determine downstream pathways involved in cystine regulation of adipogenesis, and whether cystine regulates mature human adipocyte function. The physiologic determinants of the inter-individual variability in circulating thiol concentrations and redox state also warrant investigation.

## Supplementary Information

Below is the link to the electronic supplementary material.

Supplementary file1 (DOCX 1717 KB)

## Abbreviations

bCys: Bound cysteine
CDO: Cysteine dioxygenase
CVD: Cardiovascular disease
MDA: Malondiadehyde
rCys: Reduced cysteine
rGSH: Reduced glutathione
SAA: Sulfur amino acids
SAH: S adenosyl homocysteine
SAM: S adenosyl methionine
SVF: Stromal vascular fraction
TAC: Total antioxidant capacity
tCys: Total cysteine
tGSH: Total glutathione
tHcy: Total homocysteine
