# Supplementary material for: Extracellular cystine influences human preadipocyte differentiation and correlates with fat mass in healthy adults
Source: Amino Acids. 2021 Sep 14;53(10):1623–34. doi: 10.1007/s00726-021-03071-y (PMC8521515; doi:10.1007/s00726-021-03071-y)
Supplement: Supplementary file 1 — Supplementary file1 (DOCX 1717 KB) [file 726_2021_3071_MOESM1_ESM.docx]

Protocol used for isolation and culture of human adipocyte precursor cells

Human adipose tissue samples were obtained using WaterJet-Assisted Liposuction (WAL) technique, and sampling conditions during liposuction were optimized to ensure the quality of the cells obtained. Briefly, after a small incision in the abdominal wall, an irrigation/aspiration cannula of 38 mm diameter and bear sharp tips was introduced into the adipose tissue. A flow of saline with epinephrine was used to gently detach the fat cells from the tissue, while simultaneous suction (−375 mmHg) was applied. The adipose tissue was aspirated into a 50 mL syringe under strict aseptic conditions. The time from tissue harvest to SVF isolation was <2 h.

The cell culture protocol was adapted from Bunnell et al. The lipoaspirate was washed in an equal volume of PBS + antibiotic /antimycotic, to a final concentration of (100 I.U./mL penicillin, 100 μg/mL streptomycin and 2.50 μg/mL amphotericin). For each wash, PBS was added, the bottle was gently swirled to mix, left for separation and infra-natant was aspirated and decanted. The wash of the lipoaspirate was repeated average of 6 times until the adipose layer was yellow/gold in color. The final lipid layer was digested with 0.1% collagenase type IA which was dissolved in equal volume of PBS, then filtered using 0.2 µm syringe filter. Collagenase/ lipid mixture was then placed in a shaking 37 °C water bath for approximately 1 hour. Gently the collagenase/adipose mixture was swirled every 5-10 min to allow better digestion. The adipose tissue layer become "smoother" in appearance as the digestion proceeds. After digestion the infranatant containing the Stromal vascular fraction (SVF) was aspirated into sterile falcon centrifuge tubes and equal volume of complete media was added [DMEM 4.5 g/L glucose with L-glutamine, 10% fetal bovine serum and 1% antibiotics (10,000 IU penicillin, 10,000 μg/mL streptomycin1%)] to each tube to inactivate the collagenase then centrifuged for 10 min at 300xg to collect the pellet of SVF. SVF includes mature cells such as fibroblasts, endothelial cells, smooth muscle cells, progenitors including preadipocytes, endothelial progenitor cells and vascular progenitors and stem cells as mesenchymal and hematopoietic stem cells. All SVF pellets were collected into one centrifuge tube using 10 mL PBS, passed over 100 μm cell strainer then centrifuged for 5 min at 300xg. Lysis of RBCs was done by re-suspending the SVF pellet in 5 mL of an RBC lysis buffer and incubated at room temperature for 5 min, then centrifuged for another 5 min to yield a clear SVF pellet. The cells were then counted using trypan blue counting method on a Neubar hemocytometer, seeded into 12 well plate (seeding density: 30000 cell/cm2) in growth media (DMEM 4.5 g/L glucose with L-glutamine, 10 %fetal bovine serum, penicillin/streptomycin (10,000 IU penicillin, 10,000 μg/mL streptomycin1%) in 37 °C 5% CO^2^ incubator.

Cells were monitored daily using the contrast phase inverted microscope for their growth and proliferation, and the media was changed every 2 days. When cells reach 75-80% confluence (on day 6-8 of proliferation), the adipocyte differentiation protocol was applied. Cellular differentiation and lipid accumulation were then monitored daily using a contrast phase inverted microscope, until day 8 of differentiation, when clusters of mature adipocytes filled with lipid droplets were clearly visible. In pilot experiments, preadipocyte differentiation under selected ascending concentrations of cystine were compared to differentiation under regular DMEM (Sigma-Aldrich, #D6546, https://www.sigmaaldrich.com/Formulation/DMEM6546.pdf). Regular DMEM did not increase preadipocyte differentiation and lipid accumulation beyond the effect of 50 μM cystine (Figure S1).

| **A** | **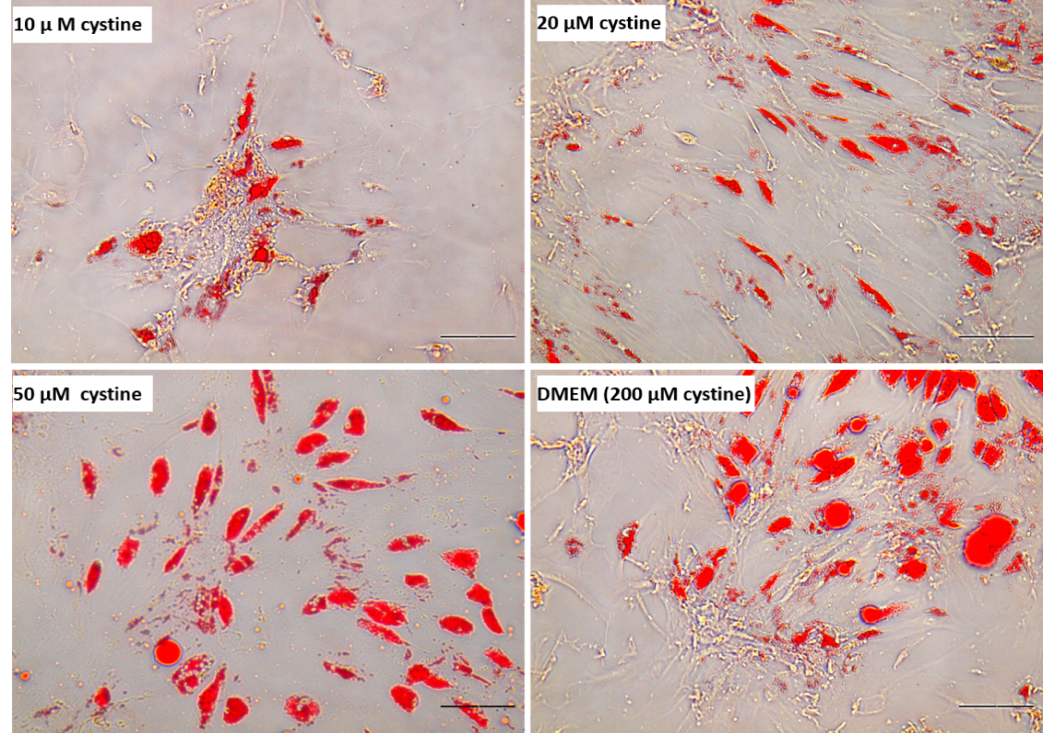** |
| --- | --- |
| **B** |  |

**Figure S1. Lipid accumulation in differentiated mature adipocytes in regular DMEM (200 μM cystine) or cystine-free medium supplemented with ascending cystine concentrations (10-50** **μM). A.** Representative images of oil red O staining for lipid content in human adipocytes under varying cystine concentrations as shown; day 8 (magnification 200x; scale bar denotes 100 µm). **B.** Quantification of the lipid-stained area on day 8 using Fiji image analysis software (NIH, Bethesda, USA). Results are mean ± SEM from 2 independent experiments, each performed in duplicates. Bars not sharing the same letter are significantly different (P ˂0.05).

**Table S1.** List of cell culture reagents

| Reagent | **Source** | **Catalogue No** |
| --- | --- | --- |
| Collagenase type IA | Sigma-Aldrich | #C9891-1G |
| ACK RBC lysis buffer | Lonza | #10-5485 |
| Penicillin and Streptomycin (P/S) | Thermo Fisher Scientific, Gibco^TM^ | #15140122 |
| Sterile Dulbecco's Phosphate Buffered Saline (1X) w/o Calcium and Magnesium (PBS) | Lonza™ BioWhittaker™ | #17512F |
| Fetal bovine serum (FBS), heat inactivated | Thermo Fisher Scientific, Gibco^TM^ | #16140071 |
| DMEM F12 media | Thermo Fisher Scientific, Gibco^TM^ | #11330032 |
| Modified DMEM (Cysteine and methionine deficient media) | Sigma-Aldrich | #D0422 |
| L-cystine. | Sigma-Aldrich | #C7602 |
| L-methionine | Sigma-Aldrich | #M5308 |
| Dexamethasone | Sigma-Aldrich | #D4902 |
| Recombinant human insulin | Sigma-Aldrich | #I91077C |
| Indomethacin | Sigma-Aldrich | #17378 |
| 3-isobutyl-1-methylxanthine (IBMX) | Sigma-Aldrich | #I5879 |
| Qiagen RNeasy Mini Kit | Qiagen | # 74104 |
| High-capacity cDNA RT kit | Life Technologies | #4374966 |
| Maxima SYBR Green/ROX kit | Thermo scientific | #K0221 |
| Primers for RT-qPCR | Biosearch Technologies | #184054 |
| Dimethyl sulfoxide (DMSO) | Sigma-Aldrich | #D2650 |
| Isopropanol | EMD Millipore | #109634 |
| 100% ethanol | EMD Millipore | #100983 |
| Paraformaldehyde | Sigma-Aldrich | #P6148 |
| Oil Red O powder | Sigma-Aldrich | #O0625 |
| Tryban blue | Sigma-Aldrich | #T8154 |
| Methylthiazolyldiphenyl-tetrazolium bromide (MTT) | Sigma-Aldrich | #M2128 |
| Centrifugation tube | Corning, Falcon^®^ | #352099 |
| 12-well-plates | Thermo Fisher Scientific | #140675 |
| Sterile Acrodisc® 0.2 μm 25 mm syringe filter | Pall life sciences | #4612 |
| 100 μm cell strainer | Corning, Falcon^®^ | #352360 |

**Table S2.** Primer sequences used in quantitative real-time PCR (qRT-PCR).

| **Primer sequences** | **GENE** |
| --- | --- |
| **Fwd:** 5`-CGAGGACACCGGAGAGGG-3`  **Rev:** 5`-TGTGGTTTAGTGTTGGCTTCTT-3` | ***PPARG1*** |
| **Fwd:** 5`-TTTTAACGGATTGATCTTTTGC-3`  **Rev:** 5`-AGGAGTGGGAGTGGTCTTCC-3` | ***PPARG2*** |
| **Fwd:** 5`-TCTCTGTTGGGGTGAAGGAC-3`  **Rev:** 5`-GCCAGGCAAATAATGTCTCC-3` | ***CDO1*** |
| **Fwd:** 5`-GTCCTTATGACAAGAACATTAGCC-3`  **Rev:** 5`-AATCAATGAAGAATGTGGTGAAG-3` | ***SCD1*** |
| **Fwd:** 5`-AAGTTGAAGCTTGAGGAGCGAGG-3`  **Rev:** 5`-GCTCGCGATGGGAACGCTGA-3` | ***PLIN1*** |
| **Fwd:** 5`-TGGCACCCAGCACAATGAA-3`  **Rev:** 5`-CTAAGTCATAGTCCGCCTAGAAGCA-3` | ***β-ACTIN*** |

**Table S3.** Correlations among plasma thiol species and body composition^1^

|  | **Age** | **Body fat%** | **rCys** | **Cystine** | **Mixed disulfides** | **bCys** | **tHcy** | **fHcy** | **tGSH** | **rGSH** | **fGSH** |
| --- | --- | --- | --- | --- | --- | --- | --- | --- | --- | --- | --- |
| **tCys** | **0.50**** | 0.17 | -0.12 | **0.45**** | **0.42*** | **0.74***** | **0.54**** | 0.34* | -0.03 | 0.07 | 0.19 |
| **rCys** | 0.26 | 0.34 | 1 | **0.64**** | **0.45**** | **-0.54**** | -0.17 | **0.48**** | -0.05 | **0.70***** | **0.63***** |
| **Cystine** | **0.46**** | **0.49***** |  | 1 | **0.84***** | -0.18 | 0.10 | **0.59**** | 0.01 | **0.46**** | **0.53**** |
| **Mixed disulfides** | **0.35*** | **0.55***** |  |  | 1 | -0.23 | 0.09 | **0.47**** | 0.06 | **0.37*** | **0.47**** |
| **bCys** | 0.23 | -0.22 |  |  |  | 1 | **0.55***** | 0.00 | -0.03 | -0.24 | -0.16 |
| **tHcy** | 0.09 | -0.03 |  |  |  |  | 1 | **0.68***** | -0.02 | 0.09 | 0.09 |
| **fHcy** | 0.18 | 0.28 |  |  |  |  |  | 1 | -0.02 | **0.52**** | **0.53**** |
| **tGSH** | -0.21 | 0.04 |  |  |  |  |  |  | 1 | 0.15 | 0.20 |
| **rGSH** | -0.06 | 0.11 |  |  |  |  |  |  |  | 1 | **0.93***** |
| **fGSH** | -0.08 | 0.12 |  |  |  |  |  |  |  |  | 1 |

1. Data are Spearman correlation coefficients adjusted for gender (age correlations) or age and gender (all other correlations) from N = 35 healthy adults. Statistically significant correlations (P <0.05) are in bold font. *P <0.05, **P <0.01, ***P <0.001. bCys, protein-bound cysteine; fHcy, non-protein bound homocysteine; mixed disulfides, all cysteine disulfides apart from cystine; rCys, reduced cysteine; rGSH, reduced glutathione; tGSH, total glutathione; tHcy, total homocysteine.
